# Identifying Ethical and Culturally Responsive Research Activities to Build Trust and Improve Participation of Black Sexual Minority Men in Pre-Exposure Prophylaxis Telehealth Clinical Trials: Qualitative Study

**DOI:** 10.2196/28798

**Published:** 2022-02-07

**Authors:** Derek T Dangerfield II, Charleen Wylie

**Affiliations:** 1 Johns Hopkins School of Nursing Baltimore, MD United States; 2 Us Helping Us, People Into Living, Inc Washington, DC United States

**Keywords:** HIV, sexual health, stigma, medical mistrust, PrEP, telehealth, medication adherence, minorities, focus groups, sexual minorities, mobile phone

## Abstract

**Background:**

Telehealth interventions could improve pre-exposure prophylaxis (PrEP) initiation and adherence in high HIV incidence groups such as young Black sexual minority men (BSMM). However, young BSMM remain distrustful of and underrepresented in clinical trials. Therefore, ethical and culturally responsive ways are needed to build trust and improve their participation in PrEP telehealth clinical trials.

**Objective:**

To bridge this gap, this study identified ethical and culturally responsive activities to build trust and improve participation of young BSMM in PrEP telehealth clinical trials.

**Methods:**

We obtained data from 7 virtual, synchronous focus groups that were conducted from April to August 2020 and consisted of 28 BSMM aged 18-34 years. Focus groups included a brief survey distributed online via Qualtrics followed by a virtual, synchronous focus group conducted via Zoom that lasted between 50 and 75 minutes. Focus groups were stratified by age (18- to 24-year-old participants and 25- to 34-year-old participants), outlined the components of an example PrEP telehealth randomized controlled trial, and included questions on domains of the study design—research motivations, study funding, recruitment activities, informed consent details, randomization, follow-up, and end of study activities. Participants were asked targeted questions regarding the ethics and trustworthiness of the study and ways in which researchers could gain their trust through the protocol used in the PrEP telehealth clinical trial.

**Results:**

The focus groups included 2 groups of 18- to 24-year-old participants and 5 groups of 25- to 34-year-old participants. The mean age of participants was 27.2 years (SD 4.4 years). Of the 28 participants, 10 (36%) reported a bachelor’s degree to be their highest completed education level and 6 (21%) reported some graduate degree or higher to be their highest completed education level. Most participants (16/28, 57%) reported that they worked full-time and that they were single or not in a committed relationship (21/28, 75%). Most participants (24/28, 86%) reported that they used at least one drug before sex in the 6 months prior to the study. All participants reported that they heard about PrEP and 36% (10/28) were current PrEP users. Overall, the focus groups yielded themes related to the impact of researcher intentions, study funding, recruitment activities, informed consent details, randomization, and study team interactions during and after the study on trust and participation in the clinical trial.

**Conclusions:**

Medical and research mistrust persists among BSMM. This study identified several ethical and culturally responsive activities to build trust and improve participation of young BSMM in PrEP telehealth clinical trials. Future studies should assess the relative impact of implementing these findings on research participation in a PrEP telehealth clinical trial.

## Introduction

To reduce disparity in the United States, it is crucial to improve participation of young Black gay, Black bisexual, and other Black sexual minority men (BSMM) in HIV pre-exposure prophylaxis (PrEP) telehealth clinical trials [1,2]. Without substantial improvement in prevention activities, BSMM have an estimated 50% lifetime risk of HIV [3]. Between 2010 and 2017, HIV incidence increased by 42% among BSMM aged 25-34 years [4]. In 2018, HIV infection in BSMM accounted for 26% of all HIV infections in gay and bisexual men in the United States; approximately 75% of newly diagnosed HIV infections in BSMM were in those under the age of 35 years [4]. Data show that PrEP substantially reduces the risk of HIV infection [5-7] and that telehealth interventions could improve PrEP initiation and adherence [8]. However, young BSMM remain underrepresented in PrEP clinical trials [9]. Increased participation in clinical trials is needed from BSMM to improve PrEP telehealth protocols.

Telehealth refers to the use of telecommunication technology to support long-distance clinical health care, health education, and health administration [10,11]. Telehealth programs are conducted remotely by a clinician via applications that are accessible on a smartphone, tablet, or computer through a video, a telephone call, or an SMS text messaging platform compliant with the Health Insurance Portability and Accountability Act of 1996 [8,11,12]. Currently, standard PrEP clinical care requires in-person visits with a clinician along with laboratory testing for HIV, sexually transmitted infections, hepatitis B, and serum creatinine levels prior to prescription [13]. However, telehealth protocols allow PrEP patients to communicate virtually with clinicians and then visit a local outpatient clinic, laboratory, or public health facility for testing [12]. Some telehealth studies mail at-home self-testing kits to patients and patients then need to return the specimens for testing prior to PrEP prescription [14]. Telehealth protocols could overcome some of the structural barriers to standard PrEP care, such as limited transportation, anticipated stigma in health care settings, and privacy concerns [15-17]. However, it has been challenging to engage young BSMM in PrEP telehealth clinical trials in part because of medical and research mistrust and the traditionally experienced stigma, discrimination in health care settings, and competing socioeconomic demands [15,18].

Medical mistrust refers to the lack of trust in the motives of and treatment by individuals and organizations associated with health care institutions [19,20]. Medical mistrust is attributed to historically limited health care access for Black Americans, including young BSMM, and maltreatment of this population by health care professionals and medical researchers [18,21]. Examples of maltreatment of Black Americans by health care professionals and medical researchers include discrimination, treatment refusal, treatment deception, and enrollment of unwilling participants in clinical experiments for research [15,21,22]. Many of these medical and research activities were supported by US racial segregation laws and a lack of established ethical research and medical guidelines [22]. The history of research and medical institutional mistreatment of racial or ethnic minority populations has impacted care satisfaction, treatment adherence, and clinical research participation among Black Americans, including young BSMM [19,23,24]. Medical mistrust is salient for BSMM [18,25,26]. Many studies have documented challenges in engaging young BSMM in HIV-prevention clinical trial research generally because of medical mistrust [24,27,28]. However, more information is needed to identify ways to improve trust and participation of this vulnerable population in PrEP telehealth clinical trial research.

To bridge this gap, this study identified ethical and culturally responsive study activities to improve participation of BSMM in PrEP telehealth clinical trials. History-based models of trust [29,30] provide a useful framework to guide this study. These models posit that trust results from cumulated, actual, or vicarious experiences and that cumulative negative experiences in society and health care settings disrupt one’s sense of safety [30]. Therefore, mistrust can increase as a safety mechanism. Although studies have identified the role of medical mistrust among BSMM in PrEP research engagement [15,18,31], more targeted data are needed to build trust, reduce mistrust, and improve participation in PrEP clinical trials for this group. Little is known about specific ways to improve trust in this population along the research process, that is, during recruitment, while obtaining informed consent, and at enrollment, follow-up, and study completion. The findings of this study could be used to design an ethical and culturally responsive PrEP telehealth intervention for BSMM.

## Methods

### Study Recruitment and Participants

We collected data from 7 virtual, synchronous focus groups that were conducted from April to August 2020 and consisted of 28 BSMM aged 18-34 years. Individuals were recruited using a combination of active and passive strategies. Active recruitment included contacting participants from other research studies who provided written consent to be contacted for future research. Passive recruitment included advertising the study on Craigslist and obtaining referrals from participants in the study. Eligibility for participation in the study was defined by the following criteria: self-identification as a Black or African American man, age between 18 and 34 years, self-report of HIV-negative status, report of oral or anal intercourse with at least one male partner in the previous 6 months, or self-identification as a gay, bisexual, queer, or non-heterosexual individual.

### Data Collection

Because of COVID-19, data collection for the focus groups was updated to a virtual, synchronous format for safety. Details of the protocol for conducting the virtual, synchronous focus group have been published [32]. Focus groups included a brief survey distributed online via Qualtrics followed by a virtual, synchronous focus group conducted via Zoom that lasted between 50 and 75 minutes. Focus groups were led by 2 experienced facilitators and were stratified by age (18- to 24-year-old participants and 25- to 34-year-old participants). One facilitator conducted the groups and recorded notes and the other scheduled the groups, recorded field notes, observed group dynamics, provided technical support for participants who had difficulty connecting to the meeting (eg, use of the wrong password), and confirmed time and attendance [32]. For data on ethical and culturally responsive ways to build trust and improve participation of BSMM in PrEP telehealth clinical trials, the focus groups outlined the components of an example PrEP telehealth randomized controlled trial and included questions on domains of the study design—research motivations, study funding, recruitment activities, informed consent details, randomization, follow-up, and end of study activities. Participants were asked targeted questions regarding the ethics and trustworthiness of the study and ways in which researchers could gain their trust through the protocol used in the PrEP telemedicine trial. Participants were given a $75 electronic gift card as compensation for their participation. All participants provided oral informed consent that was documented by the study team prior to beginning the focus group [32]. All study procedures were approved by the Johns Hopkins School of Medicine Institutional Review Board.

### Qualitative Data Analysis

Virtual, synchronous focus groups were audiorecorded using a handheld digital recorder to increase anonymity among the participants [32] and files were transcribed by a private, institutional review board–approved transcription service. The focus group facilitators reviewed all focus group transcripts and notes and developed a codebook for descriptive thematic analysis using Atlas.ti 8.4 (ATLAS.ti Scientific Software Development GmbH). Themes were identified using an adapted “pile sorting approach” [33,34]. Specifically, all quotes that were associated with specific codes in Atlas.ti 8.4 were electronically copied and pasted into an Excel sheet and organized by code. Quotes were reviewed by the lead investigator and sorted into “piles” for similarity within the Excel sheet. These piles represented the themes associated with specific focus group questions and codes. Themes were identified as patterns that were associated with specific focus group questions or expressions that provided novel responses to domains within the focus group guide [33,35,36]. To identify a range of themes, novel responses by at least one person in the group were also considered [37]. Between-group analysis was also conducted to identify potential differences in themes by age. Data presented in this study represent the range of themes related to culturally responsive ways to build trust and improve participation of BSMM in PrEP telehealth clinical trials.

## Results

### Demographic Characteristics

The focus groups included 2 groups of 18- to 24-year-old participants and 5 groups of 25- to 34-year-old participants. The mean age of the participants was 27.2 years (SD 4.4 years). Of the 28 participants, 10 (36%) reported a bachelor’s degree to be their highest completed education level and 6 (21%) reported some graduate degree or higher to be their highest completed education level. Most participants (16/28, 57%) reported that they worked full-time and that they were single or not in a committed relationship (21/28, 75%). Most participants (24/28, 86%) reported that they used at least one drug before sex in the 6 months before the study. All participants reported that they heard about PrEP and 36% (10/28) were current PrEP users (Table 1).

**Table 1:** Sociodemographic and behavioral characteristics of focus group participants (N=28).

Characteristics			Value
Age range (years)			19-34
Age (years), mean (SD)			27.2 (4)
**Sexual orientation, n (%)**			
	Homosexual, gay, or same gender loving	26 (92)	
	Bisexual	2 (7)	
**Highest education completed, n (%)**			
	Grade 11 or less	1 (3)	
	Grade 12 or GED^a^ equivalent	5 (17)	
	Some college	4 (14)	
	Bachelor’s degree	10 (36)	
	Some graduate degree or more	6 (21)	
**Employment status, n (%)**			
	Unemployed or not working	4 (3)	
	Part-time	3 (10)	
	Full-time	16 (57)	
	Other	3 (10)	
**Marital status, n (%)**			
	Single or not in a committed relationship	21 (75)	
	In a committed relationship	5 (17)	
	Married	2 (7)	
**Annual income, n (%)**			
	Less than $20,000	6 (21)	
	$20,000-$30,000	3 (10)	
	$30,000-$40,000	2 (7)	
	$40,000-$50,000	5 (17)	
	More than $50,000	10 (36)	
**Drugs used before sex in the past 6 months, n (%)**			
	Marijuana	24 (85)	
	Poppers	9 (32)	
	Ecstasy	5 (17)	
	Powder cocaine	5 (17)	
	Prescription painkillers	1 (3)	
Ever tested for HIV, n (%)			26 (92)
Ever heard of PrEP^b^, n (%)			28 (100)
Ever used PrEP, n (%)			15 (53)
Currently using PrEP, n (%)			10 (36)
Interested in PrEP injectable, n (%)			21 (75)
**Interested in PrEP telemedicine, n (%)**			
	Yes	20 (71)	
	No	6 (21)	
	Don’t know	2 (7)	
Interested in sexually transmitted infection (syphilis) PrEP, n (%)			16 (57)

^a^GED: General Education Development.

^b^PrEP: pre-exposure prophylaxis.

### Thematic Findings

Overall, the focus groups yielded themes regarding the culturally responsive ways in which researchers could build trust and improve participation in PrEP telehealth clinical trial research. Specifically, participants shared comments on how researcher intentions, study funding, recruitment activities, informed consent details, randomization, and study team interactions during and after the study could impact trust and participation. Themes regarding each domain along the exemplar clinical trial research protocol are explained.

### Intentions Behind Study Funding

Overall, participants had mixed feelings about trusting PrEP clinical trial research that was funded by pharmaceutical companies and the US government. They perceived both the government and pharmaceutical companies to be more invested in the profitability of PrEP dissemination than in the promotion of health for BSMM. They felt that an “investment in positive outcomes” could ensure greater participant safety, but they could also experience less safety because of funders’ lack of “care” for positive outcomes among Black patients. One group of 25- to 34-year-old participants shared that they had greater trust in studies funded by private foundations because of the perception that they could more easily hold these foundations accountable for any adverse events in the protocol than the government or a pharmaceutical company. Participants across groups shared that trust in the safety of the telemedicine research protocol could be gained with knowledge of the intentions of the funding source.

What do y’all think about a study like this being funded by a pharmaceutical company, versus the government, versus a private foundation?

Facilitator, 17:58

It’s all the same damn thing to me.

P4, 18:09

--I feel like pharmaceutical companies have more, you know, interest of duty kind of studies because these studies directly benefit them. And so that might make space for them to become more exploitive. Because, you know, they get something out of these studies directly versus the government. I don’t know if the money train hits them the same way as a pharmaceutical company. So the government might be less inclined promote or, organize, a very exploitive study versus pharmaceutical companies, I think.

P3, 18:31

Another group of 25- to 34-year-old participants shared the following comments:

Like, there’s money being made. And when money’s being made like that it becomes ulterior agenda. Like, even if it was something that was positive to start, it’s like, okay, well, there’s money in the research. There’s money in getting out there. There’s money in finding another way to do it. It’s--this is being pushed by money. Like, I mean, it could be a health thing. But it’s a health thing being pushed by money and money. So I think that this is still a corporation... Even if you already got it [HIV], I got a pill for that too. So it’s like, no, I don’t trust the shit, right. I don’t trust any of it anymore. And it’s not that I don’t trust the research behind it. I don’t trust the way the research is being presented.

P4, 20:59

… I’m not stupid, like, I understand there’s a business side to all of this shit. So I’m just wondering what the--even if the true intent is to help people, I’m wondering what the gag is. I’m waiting for the shoe to drop.

P2, 22:31

To that point both Green and Red’s point. Y’all got to be making a shitload of money that you can just give out $75 to us for a survey. It’s a nominal cost…And to Red’s standpoint, can there be good intentions behind it? Yes. Could the pill actually be working? Yes. But it’s kind of like, at what cost? What am I really giving away?

P3, 23:31

Yeah. Yeah. Give a piece of your liver just in case you get drunk and fuck.

P4, 25:13

These sentiments suggest a dissonance in attempts to identify altruistic intentions of funding PrEP clinical trials relative to the anticipation of maltreatment because of the profitability of positive outcomes.

### Study Recruitment: Having Black Investigative Teams and “Care”

Participants across groups shared that they would have greater trust and interest in the PrEP telehealth clinical trial if the study was led by Black researchers. They mentioned that Black researchers would provide better care during the study and interpret findings better than non-Black researchers because the participants believed that Black researchers had greater investment in the overall wellness of BSMM. When asked how researchers could gain the trust of BSMM to increase their participation in PrEP clinical trial research, participants in a 25- to 34-year-old group agreed with the man who said,

Well, I think we need to see more black queer men doing these research studies, and not necessarily the face of it but--but actually running them from the start also. So don’t just put a black face on there for, you know, image purposes or, you know, recruitment purposes, but actually have someone like us running the whole damn thing.

P4, 01:09:05

Why?

Facilitator, 01:09:34

Well, like I said, going back to like I was saying before, you know, in order for me to trust you, I have to feel like you have, uh, walked similar spaces that I have. So therefore, you can--you understand where I’m coming from when I’m telling you all this information about it.

P4, 01:09:37

When asked how non-Black researchers could gain trust, every group mentioned the need for non-Black researchers to be “involved in the community” and collaborate with community-based organizations, including the 2 groups that suggested that the race of the investigative team did not matter if they perceived that they “cared about us.” However, 1 person in a 25- to 34-year-old group said,

I feel like it’s the wrong question to ask, “how can we get black folks to trust these white researchers to come and, and, [laughter] and, and get their personal health information,” right? I think the question really should be, “how can we find more black researchers? And how can we get more black doctors? And how can we set them up for success in areas where they’re able to actually reach out with their community, right?” Like, the solution is not necessarily to place white doctors in black communities. That’s an extra barrier. And as a researcher, I don’t know why you would wanna do that.

P4, 44:28

Although participants shared an overall willingness to participate in a PrEP telehealth clinical trial led by Black investigators, they also shared that researchers generally should demonstrate care for the overall health and wellness of BSMM and not just recruit them for research.

### Informed Consent: More Clarity Regarding Adverse Effects and Data Privacy

Despite information on how a consent form would detail the risks and privacy measures involved, every group mentioned that informed consent forms should provide more explicit, thorough, and understandable details. Participants were hesitant to believe that all known adverse effects regarding PrEP would be sufficiently outlined in a consent form for a study that was classified as an experiment. Initially, the extent to which PrEP efficacy was a part of the effectiveness of the clinical trial was not clear. Moreover, participants suggested that informed consent forms should explicitly outline intentions to not cause harm to participants. For example, one young man in a 25- to 34-year-old group said that the consent form should say, “Please. Thank you. I will not hurt you,” which would be an example of the “care” for participants that BSMM suggested that researchers should demonstrate. Regarding data privacy, the older groups alluded to the need for consent forms to further describe “Who really gets access to my data?” They believed that their survey data and health history could easily be obtained by other researchers, clinicians, or pharmaceutical companies who were not a part of the study team.

### Randomization As “Not Fair”

Participants across groups generally perceived that having a computer-generated process that randomized individuals into 1 of 2 groups was fair. However, 2 participants in an 18- to 24-year-old group mentioned that they would not participate in the clinical trial if they were randomized to a telehealth treatment group because they lived with their family members and would have privacy challenges. Some 25- to 34-year-old participants expressed preferences for the telehealth treatment arm because of the perception that it would be a better experience than the standard of care. Others did not want to be in an experimental telehealth arm because they feared that their data or laboratory information could be compromised because of the virtual format.

The 25- to 34-year-old participants suggested that randomization in this study is not fair to low-resourced BSMM who may not be able to fully participate in a long-term virtual study. They agreed with the man who thought that randomization to receive telehealth did not account for a participant’s circumstance, specifically their ability to access a safe and private space to conduct a telehealth visit, to access a reliable internet connection, and to access technology.

I don’t think it’s fair--only because I think you have to think of the wholeness of the situation. If you’re randomizing someone to the telehealth group, um, again, you have you have to see what type of resources that you have to give to them.

P3, 36:24

And they can privately be able to talk, you know? Because if they don’t, it shuts out a lot of people in the community. So if we--they want it to be truly open to all people that if someone were to want to participate in the study and they are homeless, and they’re assigned to telehealth. Then we can’t guarantee them a private space or we can’t guarantee that they have reliable internet connection. We can’t guarantee that they have access to technology. Some people just do not feel comfortable sticking a needle in themselves, or whatever other, activities they need to further participate on their own. So I think that that’s why the, the randomization, I think it’s challenging, um, because there’s so much more in consideration with this community.

P3, 36:40

Although only a few participants within the groups mentioned this, group members agreed that researchers could prevent a substantial subgroup of BSMM from participating in the telehealth clinical trial by not allowing them to choose their group assignment.

### Wellness Check-Ins During Interim Visits

Participants suggested that the research team (either the staff or the principal investigator) should introduce themselves and conduct occasional wellness check-ins with participants regardless of race during interim study visits to build trust along the course of the study. They suggested that this type of communication would demonstrate “care” and investment in the overall health of BSMM. When asked to provide examples of how the research team should communicate with participants during the study, the 25- to 34-year-old participants said,

I believe that the researchers should make themselves available and present. I don’t think they necessarily be at every single transaction throughout the course of the project. But they need to have, like, check-ins or midpoints and touchpoints ‘cause that’s the whole point. So, like, just even a follow-up like, “Hey, thanks for coming out, really appreciate what you’re doing.” Just, just something to let the people know that you actually care. Like, treat them like humans because if I only see you at the beginning of the orientation and at the end when it’s all over, I’m gonna feel some kind of way because I feel like he didn’t really care about me as a person.

P4, 56:26

Okay. Does that go for a researcher of any demographic or--?

Facilitator, 56:58

Any demographic.

P4, 57:08

Yeah. I feel like you should still reach out because I feel like you have to be more personal. If you didn’t, then, I mean, I probably would never do a study with you again.

P3, 57:24

The 18- to 24-year-old participants said,

I guess email contact, maybe text messages, or maybe emails.

S2, 36:57

How often?

Facilitator, 37:06

Whatever the person is more comfortable with. I guess you just follow up, weekly or monthly or however you feel is necessary.

S2: 37:06

Yeah, I think being in touch and also letting them know that you are there for their safety and their health, that, yeah, they’re going through a study, but ultimately, it’s for their health and their well-being. Um, so being able to check on their health and their well-being, their mental health, you know, um, as well while they’re doing the study just lets them know that, “Okay, you’re committed to making sure that I'm given the support I need while helping you out.” Because we’re helping each other out, basically.

S3, 37:22

### Free PrEP and Cash Incentives as Equitable

The 25- to 34-year-old participants explicitly mentioned that PrEP should be provided for free during the study as part of the incentives because of the low prevalence of adequate insurance coverage among BSMM. They suggested that it was unreasonable to expect participants in the study to pay for the medication along with the laboratory fees associated with PrEP care and that $50 cash incentives per visit would not be sufficient or equitable. When asked if providing larger cash incentives was coercive, participants across all the groups believed that cash incentives were more equitable for their time and participation in the study. The following is an example,

So what are y’all thoughts on monetary incentives for PrEP research? Like do you think people are being exploited when you give cash?

Facilitator, 48:28

No.

P2, 48:43

No.

P4, 48:44

No. I mean it’s mutually beneficial to both parties, you know? I don’t think that that’s exploitative.

P2, 48:46

I mean people naturally want to get paid for the time, whether that be with money or some other form of incentive. So I don’t think it’s exploitative.

PS2, 48:59

Some participants also shared that participation in PrEP telehealth clinical trials was a way for some BSMM to obtain medical or financial support via clinical care and cash incentives.

Like I was saying before, a lot of people don’t have the resources to have insurance. They can’t afford insurance, some of them are only working part-time jobs. Or multiple part-time jobs that don’t pay a lot in the first place. So I think it’s kind of unfair to require that…maybe this research study is the only way I have to acquire this medication, acquire these resources because I can’t afford the insurance.

P4, 55:57

Um, I think that if you’re doing this on research, if you wanna include especially like LGBTQ people, gay people--

P3, 57:05

Young.

P2, 57:18

--then you should, you should, in that research program, find a way to provide, health care for free for at least a year. You know, some kind of--I don’t know. I know that’s a lot of work to get done, but I just feel like a lot of people in our community don’t have access to health care. So if that’s a requirement, then you’re gonna be missing out on a lot of the people that, you know what I’m saying--

P3, 57:19

Right.

P4, 57:28

### Ending Telehealth After Study as “Not Fair”

Participants across focus groups understood that PrEP telehealth resources would end with their participation and mentioned that it would be fair if the consent form explicitly stated that they would not be receiving the same resources after a specified amount of time. However, participants in the 25- to 34-year-old groups suggested that ending the convenience of PrEP telemedicine was not fair and would lower their trust and interest in future PrEP telehealth clinical trials. When asked whether not being able to have the telehealth treatment after the study ends is fair, one person said, “No. What the fuck? You can’t get me used to something for a whole year and then just take it away.” Others in the younger group said,

Not being able to have the services would definitely be, troubling if you just came to a study, and you were being helped, but now, all of a sudden, now your insurance is no longer being, paid for.

P2, 44:50

What do you think about being referred to another clinic for standard PrEP care once the study is over but you couldn’t get telemedicine?

Facilitator, 45:14

At least you will be providing them, at least referring to somewhere where you can still get your medicine, even if it’s not telemedicine.

P2: 45:24

I’m kinda thinking about my earlier comments about, like, you know, there’s an office there? I think it would kinda be hard for people who have been--for a year--had the support they needed, and they’re being cut off at the end of that year. So, I don’t know. I think it’s a tricky subject. I think that what they need to be sure is just to make sure they know at the end of this trial they may not have all of the support they used to have in that one year.

P3, 45:34

Participants viewed the study as a service provided to the community for their benefit, not necessarily an experimental treatment for a specific amount of time. They mentioned that collaborating with community-based organizations that could potentially continue similar services after the study increased their interest and trust in the study.

## Discussion

This study explored culturally responsive study activities to build trust and improve the participation of BSMM in PrEP telehealth clinical trials. Overall, PrEP telehealth was an acceptable intervention strategy among BSMM. However, source of study funding, researchers’ cultural congruence, intentions, and interactions, along with treatment assignment and ending telehealth impacted trust and study interest among BSMM. Medical and research mistrust persists in this population. The findings suggest that mistrust in PrEP telehealth clinical trials may persist because underlying issues regarding ethical clinical research conduct for minority groups have not been sufficiently addressed for BSMM. This study allows a reassessment of the traditionally acceptable domains of ethical research conduct to build trust and improve participation of BSMM in PrEP telehealth research.

The trust that BSMM have in PrEP telehealth clinical trials was assessed in part by their perception of how other and low-resourced BSMM may be treated or disregarded in the study. Concerns about the potential experiences of other in-group members is an important domain of history-based models of trust. Specifically, trust, according to this framework, is impacted by participants’ own, vicarious, or anticipated experiences [21,30]. Cumulative negative interactions with society, family members, clinicians, and researchers that are experienced or expected could outweigh the perceived benefits of PrEP telehealth clinical trials and prevent study participation. These cumulative negative interactions could exacerbate mistrust in PrEP telehealth clinical trial research because trust has generally not been established in this group. More research is needed to understand how mistrust of PrEP telehealth clinical trials results from cumulative negative social and medical experiences because BSMM have historically been low-resourced and mistreated.

This study also revealed themes that established elements of care for BSMM throughout the PrEP telehealth clinical trial protocol and greater trust in a trial led by Black investigators. Other studies have found similar themes along the lines of establishing “care” to improve trust among BSMM [24,26,32]. Studies also showed that BSMM have greater trust in and less judgement from Black clinicians and researchers [24,26,38]. This finding is important because most clinical research teams and health care providers are not culturally congruent with this population [39,40]. Having PrEP telehealth clinical trials led by Black investigators could be an important understudied structural barrier to research participation among BSMM. More work is needed to increase the number of clinical trials led by Black investigators to assess the relative impact of this preference on PrEP uptake and study participation among BSMM.

Themes regarding the fairness of randomization to telehealth treatment groups and ending telehealth services suggest that BSMM assumed that the experimental treatment arm was inherently better than the standard of care and this impacted trust in the researchers and study. The assumption that one research group in a randomized controlled trial benefits more than the other undermines the presence of equipoise and raises questions regarding the ethical considerations of PrEP telehealth randomized controlled trial protocols [41]. Some researchers suggest that some randomized controlled trials are not necessarily investigated with equipoise and that most have directional hypotheses intended to demonstrate the effectiveness of one intervention over another [41,42]. Since recent clinical trials, including PrEP telehealth studies, intend to demonstrate some positive effect of the intervention over the standard of care, there are ethical considerations regarding equipoise that remain inadequately discussed. It is reasonable to think that BSMM would assume both that telehealth treatment is better than the care given to the control group and that the benefits of the treatment do not outweigh the effort involved in participation considering their history of marginalization and minimal resources. For traditionally low-resourced groups such as BSMM, PrEP clinical interventionists should reconsider study designs that have a group that receives “better treatment.” Potentially, a single-arm pretest–posttest interventional study could be more appropriate when the assumption is that the treatment is “better” than the standard of care. Additionally, studies should request and obtain additional funding and budget to accommodate the culturally responsive activities that may be required to engage with this vulnerable population, such as providing technological devices and health care coverage for participants to sufficiently engage in the research. Traditional designs of randomized controlled trials might not be culturally responsive to the needs of BSMM and could perpetuate medical mistrust.

Importantly, we also found that information typically documented in an informed consent form (ie, minimal benefits, risks, privacy, random assignment, incentives, and end of study telehealth termination) was noted as unfair and insufficient. Guided by the Belmont Report [43], informed consent documents reflect the basic ethical principles of research conduct involving human subjects. The core tenets of the Belmont Report establish an imperative of informed consent detailing the nature of the study, benefits and risks, and randomization process and establishing participant comprehension prior to enrollment. However, data from the present study suggest that the ethical frameworks of justice and benefits within the Belmont Report [43] may require more thoughtful considerations and specificity for this subpopulation when PrEP telehealth clinical trials are conducted. Informed consent documents for PrEP telehealth clinical trial protocols may require tailoring to more adequately identify what is meant by “comprehension” and better maximize “benefits” for BSMM participants.

The limitations of this study should be considered. This study did not quantify the prevalence of medical mistrust in this sample. Additionally, this convenience sample may have been biased toward a favorable attitude to PrEP telehealth generally because of participation in the virtual, synchronous focus group. Information from participants who were unwilling or unable to participate in the virtual focus group could have impacted the range of identified themes in this study. However, data from those participants are unavailable.

Overall, this study still provided important ethical and culturally responsive considerations for improving participation of BSMM in PrEP telehealth research. Given the salience of medical mistrust in the group, future studies should quantify the prevalence of these domains in the attitudes and willingness to participate in a PrEP telehealth clinical trial among BSMM. Future research should also assess the relative impact of implementing these findings on research participation in a PrEP telehealth clinical trial.

## Abbreviations

BSMM: black sexual minority men
PrEP: pre-exposure prophylaxis

## References

[1] Nelson LE (2020). Awakening to an intersectional reality: ending the HIV epidemic in the USA starts with reducing inequities among Black MSM. J Urban Health.

[2] Wong KYK, Stafylis C, Klausner JD (2020). Telemedicine: a solution to disparities in human immunodeficiency virus prevention and pre-exposure prophylaxis uptake, and a framework to scalability and equity. Mhealth.

[3] Hess KL, Hu X, Lansky A, Mermin J, Hall HI (2017). Lifetime risk of a diagnosis of HIV infection in the United States. Ann Epidemiol.

[4] (2020). HIV and African American gay and bisexual men | HIV by group | HIV/AIDS | CDC. Centers for Disease Control and Prevention.

[5] Anderson PL, Glidden DV, Liu A, Buchbinder S, Lama JR, Guanira JV, McMahan V, Bushman LR, Casapía Martín, Montoya-Herrera O, Veloso VG, Mayer KH, Chariyalertsak S, Schechter M, Bekker L, Kallás Esper Georges, Grant RM, iPrEx Study Team (2012). Emtricitabine-tenofovir concentrations and pre-exposure prophylaxis efficacy in men who have sex with men. Sci Transl Med.

[6] (2017). PrEP | HIV Basics | HIV/AIDS | CDC. Centers for Disease Control and Prevention.

[7] Grant RM, Lama JR, Anderson PL, McMahan V, Liu AY, Vargas L, Goicochea P, Casapía Martín, Guanira-Carranza JV, Ramirez-Cardich ME, Montoya-Herrera O, Fernández Telmo, Veloso VG, Buchbinder SP, Chariyalertsak S, Schechter M, Bekker L, Mayer KH, Kallás Esper Georges, Amico KR, Mulligan K, Bushman LR, Hance RJ, Ganoza C, Defechereux P, Postle B, Wang F, McConnell JJ, Zheng J, Lee J, Rooney JF, Jaffe HS, Martinez AI, Burns DN, Glidden DV, iPrEx Study Team (2010). Preexposure chemoprophylaxis for HIV prevention in men who have sex with men. N Engl J Med.

[8] Touger R, Wood BR (2019). A review of telehealth innovations for HIV pre-exposure prophylaxis (PrEP). Curr HIV/AIDS Rep.

[9] Hoots BE, Finlayson T, Nerlander L, Paz-Bailey G, National HIV Behavioral Surveillance Study Group (2016). Willingness to take, use of, and indications for pre-exposure prophylaxis among men who have sex with men-20 US cities, 2014. Clin Infect Dis.

[10] Telehealth programs. Official web site of the U.S. Health Resources & Services Administration.

[11] Tuckson RV, Edmunds M, Hodgkins ML (2017). Telehealth. N Engl J Med.

[12] Refugio O, Kimble M, Silva C, Lykens J, Bannister C, Klausner J (2019). Brief report: PrEPTECH: a telehealth-based initiation program for HIV pre-exposure prophylaxis in young men of color who have sex with men. A pilot study of feasibility. J Acquir Immune Defic Syndr.

[13] (2018). Preexposure prophylaxis for the prevention of HIV infection in the United States-2017 Update: a clinical practice guidance. Centers for Disease Control and Prevention: US Public Health Service.

[14] Hoth AB, Shafer C, Dillon DB, Mayer R, Walton G, Ohl ME (2019). Iowa TelePrEP: A public-health-partnered telehealth model for human immunodeficiency virus preexposure prophylaxis delivery in a rural state. Sex Transm Dis.

[15] Quinn K, Dickson-Gomez J, Zarwell M, Pearson B, Lewis M (2019). "A Gay Man and a Doctor are Just like, a Recipe for Destruction": how racism and homonegativity in healthcare settings influence PrEP uptake among young Black MSM. AIDS Behav.

[16] Quinn K, Bowleg L, Dickson-Gomez J (2019). "The fear of being Black plus the fear of being gay": The effects of intersectional stigma on PrEP use among young Black gay, bisexual, and other men who have sex with men. Soc Sci Med.

[17] Rolle C, Rosenberg ES, Siegler AJ, Sanchez TH, Luisi N, Weiss K, Cutro S, Del Rio Carlos, Sullivan PS, Kelley CF (2017). Challenges in translating PrEP interest into uptake in an observational study of young Black MSM. J Acquir Immune Defic Syndr.

[18] Cahill S, Taylor SW, Elsesser SA, Mena L, Hickson D, Mayer KH (2017). Stigma, medical mistrust, and perceived racism may affect PrEP awareness and uptake in black compared to white gay and bisexual men in Jackson, Mississippi and Boston, Massachusetts. AIDS Care.

[19] LaVeist T, Isaac L, Williams K (2009). Mistrust of health care organizations is associated with underutilization of health services. Health Serv Res.

[20] Omodei MM, McLennan J (2000). Conceptualizing and measuring global interpersonal mistrust-trust. J Soc Psychol.

[21] Brandon DT, Isaac LA, LaVeist TA (2005). The legacy of Tuskegee and trust in medical care: is Tuskegee responsible for race differences in mistrust of medical care?. J Natl Med Assoc.

[22] Washington H (2007). Medical Apartheid: The Dark History of Medical Experimentation on Black Americans from Colonial Times to the Present.

[23] Arnett MJ, Thorpe RJ, Gaskin DJ, Bowie JV, LaVeist TA (2016). Race, medical mistrust, and segregation in primary care as usual source of care: findings from the exploring health disparities in integrated communities study. J Urban Health.

[24] White JJ, Dangerfield II DT, Grieb SM (2019). Methodological considerations for conducting focus groups in HIV prevention research among Black men who have sex with men. Public Health Nurs.

[25] Arrington-Sanders R, Hailey-Fair K, Wirtz AL, Morgan A, Brooks D, Castillo M, Trexler C, Kwait J, Dowshen N, Galai N, Beyrer C, Celentano D (2020). Role of structural marginalization, HIV stigma, and mistrust on HIV prevention and treatment among young Black Latinx men who have sex with men and transgender women: perspectives from youth service providers. AIDS Patient Care STDS.

[26] Dangerfield II DT, Cooper J, Heidari O, Allen S, Winder TJ, Lucas GM (2020). Nursing and health care preferences among opioid and stimulant using Black sexual minority men: an exploratory study. J Assoc Nurses AIDS Care.

[27] Magnus M, Franks J, Griffith S, Arnold MP, Goodman K, Wheeler DP, HPTN 061 Study Group (2014). Engaging, recruiting, and retaining black men who have sex with men in research studies: don't underestimate the importance of staffing--lessons learned from HPTN 061, the BROTHERS study. J Public Health Manag Pract.

[28] Benoit E, Pass M, Randolph D, Murray D, Downing MJ (2012). Reaching and engaging non-gay identified, non-disclosing Black men who have sex with both men and women. Cult Health Sex.

[29] Edmondson AC (2004). Learning from failure in health care: frequent opportunities, pervasive barriers. Qual Saf Health Care.

[30] Hammond W (2010). Psychosocial correlates of medical mistrust among African American men. Am J Community Psychol.

[31] Friedman MR, Sang JM, Bukowski LA, Chandler CJ, Egan JE, Eaton LA, Matthews DD, Ho K, Raymond HF, Stall R (2019). Prevalence and correlates of PrEP awareness and use among Black men who have sex with men and women (MSMW) in the United States. AIDS Behav.

[32] Dangerfield II Derek T, Wylie C, Anderson JN (2021). Conducting virtual, synchronous focus groups among Black sexual minority men: qualitative study. JMIR Public Health Surveill.

[33] Ryan GW, Bernard HR (2016). Techniques to identify themes. Field Methods.

[34] Ober AJ, Dangerfield II DT, Shoptaw S, Ryan G, Stucky B, Friedman SR (2018). Using a "positive deviance" framework to discover adaptive risk reduction behaviors among high-risk HIV negative Black men who have sex with men. AIDS Behav.

[35] Dangerfield II Derek T, Harawa NT, McWells C, Hilliard C, Bluthenthal RN (2018). Exploring the preferences of a culturally congruent, peer-based HIV prevention intervention for black men who have sex with men. Sex Health.

[36] Denzin KN, Lincoln YS (2011). The SAGE Handbook of Qualitative Research (SAGE Handbooks).

[37] Dangerfield II Derek T, Heidari O, Cooper J, Allen S, Lucas GM (2020). Motivations for opioid and stimulant use among drug using black sexual minority men: A life course perspective. Drug Alcohol Depend.

[38] Earl TR, Beach Mary Catherine, Lombe M, Korthuis PT, Sharp V, Cohn J, Moore R, Saha Somnath (2013). Race, relationships and trust in providers among Black patients with HIV/AIDS. Soc Work Res.

[39] Sellers K, Leider J, Harper E, Castrucci Brian C, Bharthapudi Kiran, Liss-Levinson Rivka, Jarris Paul E, Hunter Edward L (2015). The Public Health Workforce Interests and Needs Survey: The First National Survey of State Health Agency Employees. J Public Health Manag Pract.

[40] Smiley RA, Lauer P, Bienemy C, Berg JG, Shireman E, Reneau KA, Alexander M (2018). The 2017 National Nursing Workforce Survey. J Nurs Regul.

[41] Cook C, Sheets C (2011). Clinical equipoise and personal equipoise: two necessary ingredients for reducing bias in manual therapy trials. J Man Manip Ther.

[42] Gifford F (2001). Uncertainty about clinical equipoise. Clinical equipoise and the uncertainty principles both require further scrutiny. BMJ.

[43] National Commission for the Protection of Human Subjects of Biomedical and Behavioral Research (1979). The Belmont Report. U.S. Department of Health & Human Services Office for Human Research Protections.

